# Federal Funding in Emergency Medicine: Demographics and Perspectives of Awardees

**DOI:** 10.5811/westjem.2019.12.45249

**Published:** 2020-02-24

**Authors:** Peter R. Chai, Stephanie Carreiro, Brittany P. Chapman, Edward W. Boyer, Kelli N. O’Laughlin

**Affiliations:** * Brigham and Women’s Hospital, Department of Emergency Medicine, Boston, Massachusetts; † Harvard Medical School, Department of Emergency Medicine, Boston, Massachusetts; ‡ The Fenway Institute, Boston, Massachusetts; § Massachusetts Institute of Technology, The Koch Institute for Integrated Cancer Research, Cambridge, Massachusetts; ¶ University of Washington, Department of Emergency Medicine, Seattle, Washington; || University of Massachusetts Medical Center, Department of Emergency Medicine, Division of Medical Toxicology, Worcester, Massachusetts; # University of Washington, Department of Global Health, Seattle, Washington

## Abstract

**Introduction:**

Emergency physicians face multiple challenges to obtaining federal funding. The objective of this investigation was to describe the demographics of federally-funded emergency physicians and identify key challenges in obtaining funding.

**Methods:**

We conducted a retrospective database search of the National Institutes of Health (NIH) Research Portfolio Online Reporting Tool (NIH RePORTER) to collect data regarding the distribution and characteristics of federally-funded grants awarded to emergency medicine (EM) principal investigators between 2010–2017. An electronic survey was then administered to the identified investigators to obtain additional demographic data, and information regarding their career paths, research environment, and perceived barriers to obtaining federal funding.

**Results:**

We identified 219, corresponding to 51 unique, mentored career development awardees and 105 independent investigators. Sixty-two percent of investigators responded to the electronic survey. Awardees were predominantly White males, although a larger portion of the mentored awardee group was female. Greater than half of respondents reported their mentor to be outside of the field of EM. The most common awarding institution was the National Heart Lung and Blood Institute. Respondents identified barriers in finding adequate mentorship, time to gather preliminary data, and the quality of administrative support.

**Conclusion:**

The last five years have showed a trend toward increasing grants awarded to EM investigators; however, we identified several barriers to funding. Initiatives geared toward support and mentorship of junior faculty, particularly to females, minorities, and those in less heavily funded areas of the country are warranted.

## INTRODUCTION

Research grants awarded from federal sources such as the National Institutes of Health (NIH) remain a leading measure of success in academic medicine. Clinician scientists pursing a research career, including emergency physicians (EP), often pursue a career development award (ie, K grants) as a bridge to independent funding status (ie, R-series awards).^1^ Career development awards are considered prestigious to the investigator as well as to his or her sponsoring department, but they require a significant investment of time from the trainee and mentorship team.^2^ Successful submission of a federal career development grant may require up to two years of preparation, which includes finding a committed mentorship team, crafting a research and training plan, initiating pilot data collection, generating a sufficient number of publications to demonstrate commitment to academic practice, and undergoing a rigorous review process. During this time, emergency medicine (EM) faculty are faced with significant barriers including clinical commitments, administrative obligations, and time required for mentorship meetings and grant writing.^3^ They also face the challenges associated with the transition into junior faculty.^3^ Compared to other specialties, EPs submit the fewest mentored career awards (K grants), have the third lowest success rate (60% funded), and submit the fewest grants per faculty size.^2^,^4^

EPs are well positioned to make meaningful contributions to research given their breadth of clinical expertise across a wide spectrum of disease, unique window into the communities where they practice, and natural collaboration with multiple clinical disciplines. Despite this, a 2006 Institute of Medicine report demonstrated that few NIH training grants were awarded in emergency departments (ED).^5^ Recognizing the immediate importance of training future clinician-researchers in EM led to the creation of the Office of Emergency Care Research (OECR) in 2012 to coordinate research in this rapidly growing space. 6,7

From 2011 to 2014, emergency care research made up only 0.7% of NIH spending on new grants.^8^ In response, the Society for Academic Emergency Medicine (SAEM) and the American College of Emergency Physicians (ACEP) recommended four key strategies to increase the pipeline of federally funded emergency care researchers: 1) promote research as a viable career trajectory; 2) identify the availability of senior mentors; 3) understand the process of applying for NIH funding as a financial investment; and, 4) build a supportive culture that fosters research.^9^ Currently, several EM-based, NIH-funded training programs (eg, T32, K12) provide structured mentorship and funded protected time that allow junior academicians to gather preliminary data in support of subsequent investigations.^10^ These programs, in addition to increased support for a research career path, have resulted in 1.7% of funded NIH grants attributed to EM faculty from 2008 to 2017.^11^ Although improved, these statistics indicate that EM researchers still receive relatively few NIH grants in comparison to other specialties.

The NIH Research Portfolio Online Reporting Tool (NIH RePORTER) is a publicly available database and a tool that can be used to better understand NIH funding related to EM faculty in the United States.^12^ Outside of official NIH reporting, however, limited data exists to assess variables that are important to achieving NIH funding, such as the clinical specialty of mentors, the availability of protected time, and access to department-funded research infrastructure. Knowledge regarding the prevalence of these important variables among NIH-funded EM investigators may be useful for individuals seeking a research career to help in selecting academic positions, and for EDs working to enhance research among their faculty.

**Population Health Research Capsule**

What do we already know about this issue?

Emergency physicians (EP) face challenges in obtaining funding, finding mentors, and managing the balance between research and clinical work.

What was the research question?

To define the demographics of federally funded EPs and their barriers to obtaining funding.

What was the major finding of the study?

Most 2010–2017 awardees were White males, although women got more mentored career grants. EPs still experience difficulty finding adequate mentors.

How does this improve population health?

Increased support for the EP researcher, especially for women and minorities, remains important in growing the body of federally funded EPs.

The goal of our study was to use both NIH RePORTER data and individual surveys to describe the following: 1) the distribution and characteristics of NIH grants awarded to EPs; 2) the principal investigator (PI) characteristics and resources accessed for these successful applications; and, 3) perceived facilitators and barriers to the NIH grant funding process from the PI’s perspective.

## METHODS

### Study Design and Selection of Participants

This investigation was composed of two parts. Part one was a retrospective database search using the NIH RePORTER to collect data regarding federally funded grants awarded to EM PIs. We included individuals who were funded by the Agency for Healthcare Research and Quality and the Centers for Disease Control and Prevention, as these federal grants are included in NIH RePORTER. Part two was a survey-based investigation that queried the NIH-funded EM PIs identified in part one to obtain additional demographics as well as information on their career paths, research environment, and perceived barriers to applying for funding. The study protocols for parts one and two were deemed exempt by the human subjects institutional review board by the Partners Human Research Committee.

### Part I: NIH RePORTER Data Abstraction

A list of relevant NIH-funded research projects was curated from the NIH RePORTER search function using the following search criteria: 1) funding received between fiscal years 2010–2017 (10/01/10 – 09/30/17); and 2) department type listed as EM. One author (BPC) manually reviewed this list and removed projects funded in this period without a start date in this timeframe. We extracted variables from all remaining records including the PI’s gender and academic rank, grant mechanism (eg, K- or R-series), PI contact information, start year of the grant, total years of funding awarded to the grant, and NIH funding institute. We also recorded the geographic location of the PI’s primary institution.

### Part II: EM PI Survey

Using data extracted from part one, we stratified the identified EM PIs into two cohorts: 1) “mentored PIs” with career development awards (eg, K08, K12, K23); and 2) “independent PIs” with independent research grants (eg, R01, R34, R21). We included individuals supported by a cooperative agreement (U-mechanism) or mid-career mentoring award (K24) with the independently funded cohort. Individuals who were listed as having both a K grant and a subsequent or parallel R grant were included only in the independent PI group. We electronically distributed surveys to all of the PIs identified, based on these two cohorts.

We designed two separate surveys to answer key questions about the demographics of NIH-funded investigators, and the relationship of their research area to EM. Surveys were created through an iterative process among the study team. The study team identified themes surrounding funding, research topic, and mentorship and then drafted several questions surrounding these concepts. Next, the study team selected questions that were clear and piloted these on non-study team EP investigators to ensure clarity of the questions. These final surveys were then administered to mentored PIs and independent PIs. The mentored PI survey (Appendix 1) included questions on demographic data (gender, ethnicity, race, and academic rank) and on research focus and environment (including mentor’s academic department, administrative support, pre- and post-award grant administrative support, and average monthly hours worked in the ED during the award period).

The independent PI survey (Appendix 2) was designed to collect demographic data (gender, age, ethnicity, race, academic rank), information about research career (prior K award funding), research focus, and research environment. Both surveys included open-ended questions asking about barriers that EM PIs faced in obtaining career development awards.

### Data Management

We used the Research Electronic Data Capture (REDCap) tool to capture and manage all study data including surveys.^13^ Reminder emails were sent on days 10, 20, and 30 to individuals who had not started or who had partially completed the survey; the survey link expired on day 31. All study communications were sent via the REDCap database in survey mode.

### Data Analysis

For quantitative data, we determined basic descriptive statistics for sociodemographic variables, grant characteristics, and institution characteristics. We calculated percentages for categorical variables, and calculated medians with interquartile ranges for continuous variables. We constructed heat maps using key regions of the United States defined by the US Census Bureau to explore the geographic distribution of identified grants. We analyzed all quantitative data using STATA version 15.1 (StataCorp LLC, College Station, TX).

For qualitative data, we used a conventional content analysis approach to understand participants’ experiences.^14^ In conventional content analysis, coded categories are taken from the text allowing us to derive information from responses without preconceived categories. Two analysts (PRC, SC) reviewed all of the responses and inductively derived codes based on content similarity within the text. We revised groupings using an iterative process of content review and returning to the data. Analysts debated discrepancies until consensus was achieved.

## RESULTS

### Part I: NIH RePORTER Results

Over the seven-year study period, we identified 219 grant awards from NIH RePORTER records that met inclusion criteria (Table 1), which were awarded to 156 unique individual investigators. A majority of grants (N = 162, 74%) were awarded to males. Grants were fairly evenly distributed by academic rank, with 39% of PIs listed as professor, 26% as associate professor level, and 31% as assistant professors. The most common awarding NIH institutions were the National Heart Lung and Blood Institute (26%), the National Institute of Neurological Disorders and Stroke (13%), and the Agency for Healthcare Research and Quality (12%). An average of 31 NIH awards were obtained annually from fiscal year 2010–2017, with a steady upward trend from 2013–2017. The most commonly identified mechanisms were R01 (29%), K23 (6%), K12 (5%), and R03 (5%).

**Figure 1:**
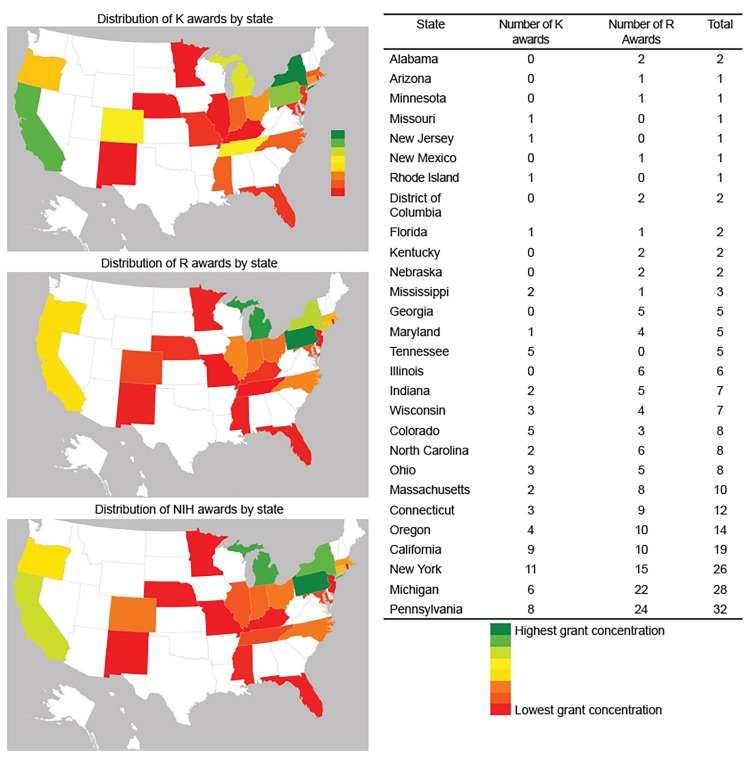
Geographical distribution of NIH awards across the United States.

We identified a greater clustering of grants awarded to PIs located in the Midwest and New England (Figure 1) than within other regions of the country. Additionally, mentored career development awards and independent investigator awards appeared to cluster together in the same regions.

### Part II: Survey results

We sent electronic surveys to 51 unique mentored PIs identified in Part I with a 69% response rate (N = 35), and to 105 independent PIs identified from Part I with a 58% response rate (N = 61).

### Demographics, Investigator and Research Characteristics

The majority of mentored PIs were male (N = 20, 59%), White (N = 28, 82%), and at the rank of assistant professor (N = 24, 69%) (Table 2). Overall, the proportion of grants awarded to women over the study period was stable. Mentored PIs reported their primary mentors were in EM (N = 11, 32%), cardiology (N = 5, 15%), internal medicine (N = 4, 12%), and infectious disease (N = 2, 6%). Most research conducted under the mentored career development mechanism was focused in EM (N = 24, 71%). Only three (9%) participants did not have grants administration support within their department. Participants reported that they worked an average of 48 clinical hours per month during the time of their K award.

Of the independent PI respondents, the majority identified as male (N=46, 77%) and White (N = 48, 80%). Most participants (N = 38, 63%) did not have a mentored career development award prior to obtaining independent funding. Participants who received a K award reported working an average of 41 clinical hours per month during their K award period. Independent investigators commonly identified having a mentor within EM (N = 22, 40%) or internal medicine (N = 12, 22%).

### Barriers to obtaining mentored career development awards

When asked about barriers to obtaining mentored career development awards, 88% (N = 30) of K survey respondents and 28% (N = 19) of R-funded survey respondents provided answers. Commonly identified barriers were similar between both groups: 1) finding appropriate mentorship; 2) having appropriate time to prepare and submit a K award; and, 3) lacking a robust administrative infrastructure to support NIH awards (Table 3). With respect to mentorship, participants specifically reported significant barriers in finding adequate mentors in EM. Twenty-four participants reported that they sought mentorship outside of EM in disease-specific areas; many cited the structure of disease-based NIH awarding institutes as a driving factor. Participants also reported that a lack of mentorship at times prevented them from learning about the value of career development awards and detracted from their perceived ability to pursue this line of funding

Participants also reported difficulty in finding time away from clinical and administrative commitments to prepare and submit a K application. The competing priorities of clinical work, teaching, and completing administrative tasks interfered with the time needed to meet mentors, generate sufficient publications, and prepare the grant application. Additionally, participants reported variability in the degree of administrative support their departments provided in applying for NIH awards.

## DISCUSSION

This investigation demonstrates that despite recent efforts to foster NIH-funded research in EM, there remain critical barriers to successful funding, particularly for early-career investigators. However, for EM to achieve its maximum potential impact in research, we should focus on providing investigators with mentorship, protected time, and grants of administrative support to enhance success.

Almost half (41%) of K-funded respondents identified as female compared to 23% of independently funded investigators. The proportion of female EM investigators funded under a K mechanism is higher than reported in other specialties such as surgery and anesthesiology, yet the total number of grants overall awarded to women has not changed.^15^ This may demonstrate success of recent initiatives to promote research among female junior faculty. While the higher proportion of female-mentored PIs compared to independent PIs may reflect a trend toward gender equity, an alternative explanation may be that female investigators are not successfully transitioning to independent funding status. This is concerning because overall NIH data and investigations within other specialties demonstrate increasing parity among men and women who transition from mentored to independent awards.^16^,^17^,^18^ Among EM investigators, the transition rate from mentored to independent investigator is approximately 40%, yet the proportion of women who successfully complete this transition is unknown.^19^ Our data suggests that continued efforts to support women, especially during the end of the mentored award period, is needed within EM to improve gender equity among independent researchers

While initiatives such as junior faculty development programs and female-specific mentoring may increase the number of women pursuing careers in research, a focus on continued mentorship and support for female faculty as they transition from mentored research to independent funding status may help increase the number of independently-funded female investigators in EM. The difficult transition from mentored grants to independent investigator status should not be ignored; support from academic departments at the early career phase should occur in synergy with support at the transition to independent funding.^25^

In parallel with promoting careers in NIH-funded research for women, we should also focus on increasing racial diversity. Our survey respondents of both training and independently funded NIH awards overwhelmingly identified as White. Initiatives that promote research careers among women, as well as among racial and ethnic minorities, should continue to be high priorities for EM.^20^

We found that mentored career-oriented grants and independent investigator awards tended to cluster by geographic region, which is consistent with prior literature.^21^ One explanation for this phenomenon is that independently funded investigators attract other EPs who initiate research careers. Departments with a strong research division also offer a large professional network and access to resources that benefit junior investigators. Finally, EDs with senior investigators may indicate a commitment by the department to a career in research, thereby providing strong grants support, mentorship, and even seed funding to junior investigators. Departments with focused mentoring programs result in increased NIH funding success, increased number of publications, and higher levels of perceived success.^22^ Academic EDs that may not have NIH-funded scientists on faculty, specifically in rural areas, may have important and fundable priorities to study. Targeted interventions that extend resources to traditionally less research heavy institutions (eg, seed funding for preliminary data, and a network of available mentors willing to work with new investigators at remote sites) can create equitable opportunities for research careers across the country.

Our qualitative survey data suggests that lack of mentoring, time, and grants infrastructure hamper successful NIH award applications among EM investigators. Less than half of mentored PIs identified that their primary mentor was in EM. Participants reported that they sought mentorship outside of EM due to a lack of NIH-funded EM faculty and lack of mentorship within a disease state. Continued promotion of the NIH career track and increasing numbers of independently funded EM faculty will hopefully increase the availability of those who can serve as mentors for junior faculty. Comprehensive mentorship programs as part of faculty development programs or department-initiated research mentoring can help create an environment that promotes and supports federally funded research.^3^,^23^

Participants also commented on the difficulty of preparing a K award in the context of transitioning to junior faculty status and completing clinical and administrative duties within EM. Balancing the unpredictable clinical schedule of an EP with time needed to meet and establish mentoring plans, publish manuscripts, generate preliminary data, and prepare grant submission materials was a barrier reported by participants in this study. Finding protected time to write and conduct preliminary research is especially difficult for junior faculty, yet the value of dedicated time early in one’s career cannot be underestimated.^24^ The barriers we identified are similar to those addressed by a joint SAEM/ACEP research committee.^9^ While many institutions have junior faculty development programs geared toward supporting early-career physicians, EDs must continue to consider the importance of dedicating time and funding to their junior faculty to boost and support research careers during this vulnerable stage. Peer mentoring groups should also be considered as a supplement, although they cannot replace senior mentorship.^3^

## LIMITATIONS

Our primary data source from Part One (NIH RePORTER) is limited and would not capture grants or EM PIs listed under a different department, or those listed under institutional career development awards (eg, K12, KL2 mechanisms). We do not know how many potential EM investigators would have been missed through our query using NIH RePORTER. The constraints of our search mechanism and the nature of our study population led to a small sample size, limiting our analysis. The low response rate on our survey component creates potential for missing and/or biased data. Finally, some survey participants reported they had limited time to answer the survey questions, and as a result, did not provide answers to all questions.

## CONCLUSION

Our review of funded EM grants through the NIH RePORTER system and individual surveys of mentored and independent investigators demonstrated several positive trends, including increasing gender diversity in early-career mentored grants, yet there continue to be important areas for improvement such as access to mentorship, grants infrastructure, and dedicated time early in a career to develop important research opportunities. Continued support for research as a career path and mentoring of early-career physicians is important in growing the cadre of EM researchers. Future work, such as longitudinal studies to evaluate individual characteristics associated with success and interventions geared toward increasing mentorship and support, are needed.

## Supplementary Information





## Figures and Tables

**Table 1:** Grant characteristics from NIH RePORTER (N=219).*

Principal Investigator Demographics		
Sex	Female	57 (26.%)
	Male	162 (74%)
Rank	Fellow	2 (1%)
	Instructor	5 (3%)
	Assistant Professor	55 (31%)
	Associate Professor	47 (26%)
	Professor	70 (39%)

Award characteristics		

Activity Code**	K08	9 (4%)
	K12	11 (5%)
	K23	34 (16%)
	K24	3 (1%)
	R01	63 (29%)
	R03	11 (5%)
	R18	8 (4%)
	R21	23 (11%)
	R34	6 (3%)
	R56	5 (2.3%)
	U01	10 (5%)
	U24	9 (4%)
Start Year	2010	29 (13%)
	2011	21 (10%)
	2012	21 (10%)
	2013	18 (8%)
	2014	22 (10%)
	2015	28 (13%)
	2016	37 (17%)
	2017	43 (20%)
Admin Institute***	NHLBI	57 (26%)
	NINDS	28 (13%)
	AHRQ	26 (12%)
	NIA	19 (9%)
	NIDA	17 (7.8%)
	NICHD	11 (5.0%)
	NIDDK	9 (4%)
	NIAAA	8 (4%)
	NIGMS	7 (3%)
	NIMH	7 (3%)
	NIAID	4 (2%)
	FIC	3 (1%)
	NCIPC	3 (1%)
	NIMHD	3 (1%)
	NINR	3 (1%)

* These data include multiple grants awarded to the same individual.

** Others, < 1 % N = 1–2 UM1, UH4, UH2, U34, U10, T35, T32, T15, RC4, RC1, R35, R25, R24, KL2, K99, K01, G20, F32, F31.

*** Others with < 1 % (NCATS, NCRR, NEI, NIEHS, NIOSH, NLM, ONCHIT).

*NHLBI*, National Heart, Lung and Blood Institute; *NINDS*, National Institute of Neurological Disorders and Stroke; *AHRQ*, Agency for Healthcare Research and Quality; *NIA*, National Institute of Aging; NIDA: National Institute on Drug Abuse; *NICHD*, Eunice Kennedy Shriver National Institute of Child Health and Human Development; *NIDDK*, National Institue of Diabetes and Digestive and Kidney Diseases; *NIAAA*, National Institute on Alcohol Abuse and Alcoholism; *NIGMS*, National Institute of General Medical Sciences; *NIMH*, National Institue of Mental Helath; *NIAID*, National Institute of Allergy and Infectious Diseases; *FIC*, Fogarty International Center; *NCIPC*, National Center for Injury Prevention and Control; *NIMHD*, National Institute on Minority Health and Health Disparities; *NINR*, National Institute of Nursing Research.

**Table 2:** Principle investigator survey data.

	K Awardees (N=35)	R Awardees (N=61)
Demographics		
Sex		
Female	14 (41%)	14 (23%)
Male	20 (59%)	46 (77%)
Age (median, IQR)	41 (37, 45)	47 (43, 56)
Ethnicity		
Hispanic/Latino	1 (3%)	4 (7%)
Not Hispanic/Latino	32 (97%)	50 (89%)
Other	0 (0%)	2 (4%)
Race		
Asian	5 (15%)	10 (17%)
Black or African American	1 (3%)	1 (2%)
White	28 (82%)	48 (80%)
Multi-racial	0 (0%)	1 (2%)
Rank at K award		
Instructor	4 (11%)	
Assistant Professor	24 (69%)	
Associate Professor	5 (14%)	
Professor	2 (6%)	
Prior K award		
No	N/A	38 (63%)
Yes	N/A	22 (37%)
Research focus and environment		
Research EM focused		
No	10 (29%)	
Yes	24 (71%)	
Mentor’s academic department		
Emergency Medicine	11 (32%)	22 (40%)
Other*	14 (40%)	16 (32%)
Cardiology	5 (15%)	1 (2%)
Internal Medicine	4 (12%)	12 (22%)
Any grant administrator support		
No	3 (9%)	6 (10%)
Yes	31 (91%)	53 (88%)
Grants administrator availability		
Both Pre- and Post-Award	27 (87%)	52 (98%)
Don’t know	1 (3%)	1 (2%)
Pre-Award Only	1 (3%)	0 (0%)
Post-Award Only	2 (6%)	0 (0%)
Clinical hours worked per month during K, median (IQR)	48 (32, 55)	41 (32, 55)

* Includes Psychiatry, Surgery, Behavioral Science, and “other”.

**Table 3:** Qualitative themes for barriers to career development awards.

Theme	Illustrative response
Lack of mentorship	“I avoided pursuing a K for a long time due to lack of perceived available mentors and a desire for more research funding and less funding for training.” “Finding a topical mentor was difficult; I had to go outside my institution to find one.” “None of my official K award mentors are from EM. I have a joint appointment in another department that has more NIH-funded researchers and research infrastructure, which was important for me to be successful when applying for my K.”
Managing time to prepare grants	“Clinical hours are a struggle. I currently need to further buy down my time in order to do the research.” “Initial buy down of clinical time to write the K23 award was the biggest barrier for me.”
Administrative support	“Navigating the complex NIH system with little administrative support was a huge pain.” “Lack of research infrastructure in the department made non-research related submission details difficult.” “I did not have grant administration support when I got my K so I had to do all of the pre-award stuff on my own.”
