# Supplementary material for: Federal Funding in Emergency Medicine: Demographics and Perspectives of Awardees
Source: West J Emerg Med. 2020 Feb 24;21(2):304–12. doi: 10.5811/westjem.2019.12.45249 (PMC7081857; doi:10.5811/westjem.2019.12.45249)
Supplement: Supplementary file 1 [file wjem-21-304-s001.pdf]

NIH Funding in EM: K Funded Investigators

Thank you for participating in the survey for IRB Protocol# 2017P001202. Your participation in this survey and study is entirely voluntary. We will not be collecting any protected health information, and the information you provide here will remain de-identified.

Gender	◯ Male<br>◯ Female
Age	__________________________
Race	◯ Asian<br>◯ Black or African American<br>◯ American Indian or Alaska Native<br>◯ Native Hawaiian or Other Pacific Islander<br>◯ White<br>◯ Other
Please specify	__________________________
Ethnicity	◯ Hispanic/Latino<br>◯ Not Hispanic/Latino<br>◯ Other
Please specify	__________________________
Where did you do your emergency medicine residency?	__________________________<br>(If not applicable, please type "N/A")
What was your academic rank at the time your grant was awarded?	◯ Clinical Instructor<br>◯ Member of the Faculty<br>◯ Assistant Professor<br>◯ Associate Professor<br>◯ Professor<br>◯ Other
Please specify	__________________________
How many years out of residency were you when you were awarded your K?	__________________________<br>(If not applicable, please type "N/A")

The next few questions will be about your mentoring team for your K.

Which department is your primary mentor in?

◯ Internal Medicine
◯ Surgery
◯ Cardiology
◯ Infectious Disease
◯ Emergency Medicine
◯ Psychiatry
◯ Behavioral Science
◯ Other

Please specify

____

Is your research emergency medicine focused?

◯ Yes
◯ No

Thank you for your participation so far. This is the last section of the survey where we will ask you about your grants administration and clinical support.

Does your department have a grants administrator?

- ◯ Yes
- ◯ No
- ◯ N/A

Does your grants administrator provide pre and post award support?

- ◯ Pre award only
- ◯ Post award only
- ◯ My grants administrator provides both pre and post award support
- ◯ I don't know

How many clinical hours did you work per month during your K award period?

____
(Hours/Month)

What barriers did you experience in obtaining your K award?

____

Please list emergency medicine investigators that you know who are NIH funded:

____
